# An adult case of severe life-threatening *Mycoplasma pneumoniae* pneumonia due to a macrolide-resistant strain, Japan: a case report

**DOI:** 10.1186/s12879-019-3846-1

**Published:** 2019-02-28

**Authors:** Munehiro Matsumoto, Kentaro Nagaoka, Masaru Suzuki, Satoshi Konno, Kei Takahashi, Taichi Takashina, Nobuhisa Ishiguro, Masaharu Nishimura

**Affiliations:** 1 Department of Respiratory Medicine, Faculty of Medicine and Graduate School of Medicine, Hokkaido University, Sapporo, Japan; 2 Division of Respiratory Medicine, Iwamizawa Municipal General Hospital, Iwamizawa, Japan; 3 Department of Infection Control, Hokkaido University Hospital, Sapporo, Japan

**Keywords:** *Mycoplasma pneumoniae*, Pneumonia, Macrolide-resistance, Adult

## Abstract

**Background:**

Until now, the prevalence of macrolide-resistant *Mycoplasma pneumoniae* (MP) infection among adult patients has been low, and severe MP pneumonia due to a macrolide-resistant strain has seldom been reported. Here, we describe a rare case of severe life-threatening MP pneumonia due to a macrolide-resistant strain in an adult, which was finally treated with fluoroquinolone and tetracycline after failed treatment with macrolide and corticosteroid.

**Case presentation:**

A 39-year-old apparently healthy woman complained of fever and productive cough. Three days after onset, she was admitted to a local general hospital. On admission, her vital signs were stable except for high-grade fever. The patient’s chest X-ray and chest computed tomography images revealed subsegmental consolidation in her right lower lobe. Treatment with ampicillin/sulbactam, and azithromycin were initiated under a clinical diagnosis of community-acquired pneumonia. After treatment initiation, her fever had not subsided, and the pulmonary lesion had extended to the entire lower lobe. Thus, treatment with prednisolone as steroid pulse therapy was initiated from clinical day 7. However, neither her symptoms nor her pulmonary lesion improved; therefore, she was transferred to our hospital for further examination and treatment. On admission (clinical day 14), her indirect hemagglutination titer for MP was elevated at 1:2560, and bronchoalveolar fluid examination yielded positive results for the mycoplasma antigen. Based on these clinical findings, we confirmed a case of severe life-threatening MP pneumonia. Since her respiratory condition was extremely severe, we initiated levofloxacin and tetracycline. Two days later (clinical day 16), her fever, malaise, and hypoxia resolved, and her pulmonary lesions had significantly improved.

Further molecular identification yielded the DNA of MP from her bronchoalveolar fluid, and mutation of A2063G in the 23S rRNA gene was revealed. Based on these results and the clinical course, we confirmed our case as severe MP pneumonia due to a macrolide-resistant strain.

**Conclusion:**

More awareness is needed on the emergence of macrolide-resistant MP infection in adults, because severe infection could develop despite initial treatment with macrolide and steroid therapy, which are generally considered as standard therapy for MP.

## Background

*Mycoplasma pneumoniae* (*M. pneumoniae*; MP) is a major cause of community-acquired pneumonia in children and adults [1]. MP pneumonia (MPP), often described as a self-limiting disease, is typically mild and cured without medication. However, approximately 0.5–2% of all MPP cases is known to present a fulminant course with severe complications such as respiratory failure [2]. For the treatment of severe life-threatening MPP, early administration of anti-mycoplasma drugs, such as macrolides (erythromycin, clarithromycin, and azithromycin), and corticosteroids has been recognized as advantageous [2–4]. Recently, the prevalence of macrolide-resistant MP has emerged in several countries, including Asia, Europe, and the United States [5–7]. More than 60% of MP strains among pediatric patients in Japan have been reported to possess a macrolide-resistance mutation [8]. In contrast, the prevalence of macrolide-resistant MP infection among adult patients has, thus far, been considered low [9], and severe MPP due to a macrolide-resistant strain has rarely been reported. Here, we describe a case of severe life-threatening MPP due to a macrolide-resistant strain (23S rRNA gene A2063G) in an adult, which was finally treated with fluoroquinolone and tetracycline after failed treatment with macrolide and corticosteroid therapy.

## Case presentation

**Fig. 1:**
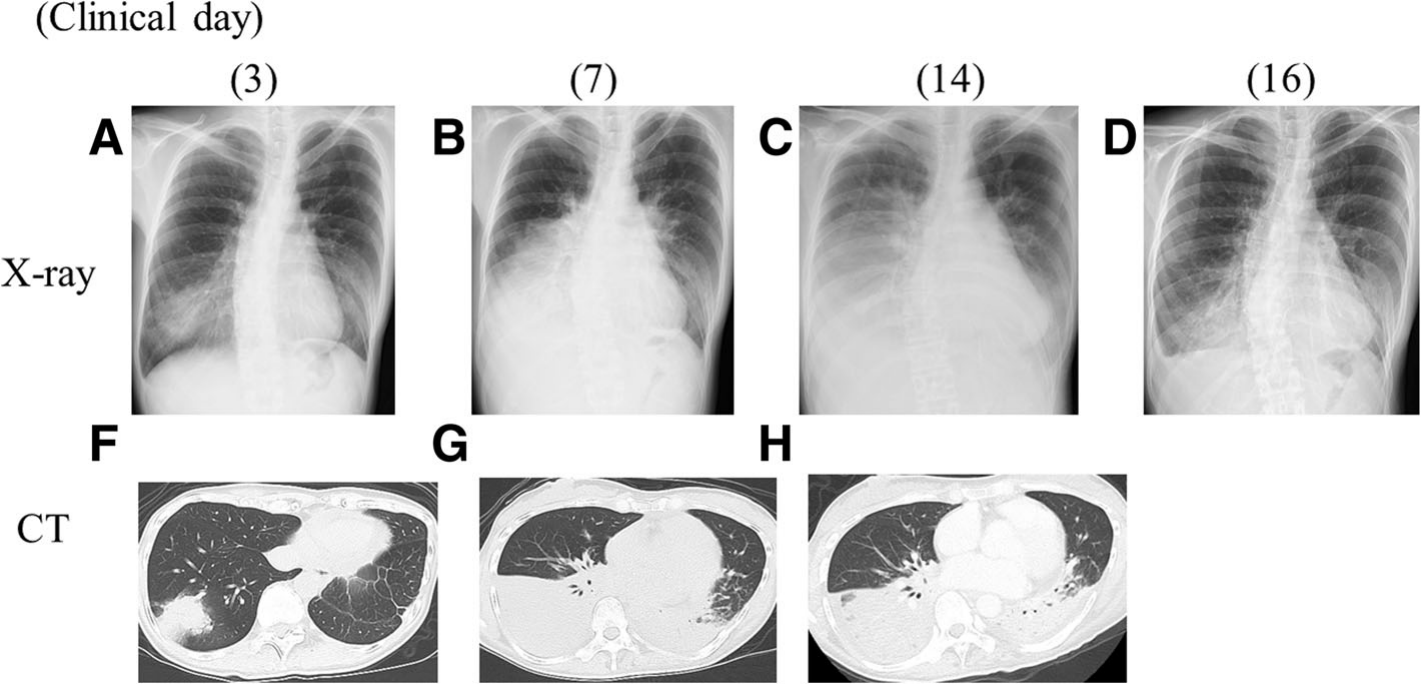
Chest X-ray image (A, B, C and D) and chest CT image (F, G and H) of the thorax. 10 days before admission to our hospital (clinical day 3), the chest images revealed sub-segmental infiltration in the right lower lobe, which enlarged to bilateral lower lung field from clinical day 7 to 14. After switching the antibiotics, the chest images improved until clinical day 16

**Fig. 2:**
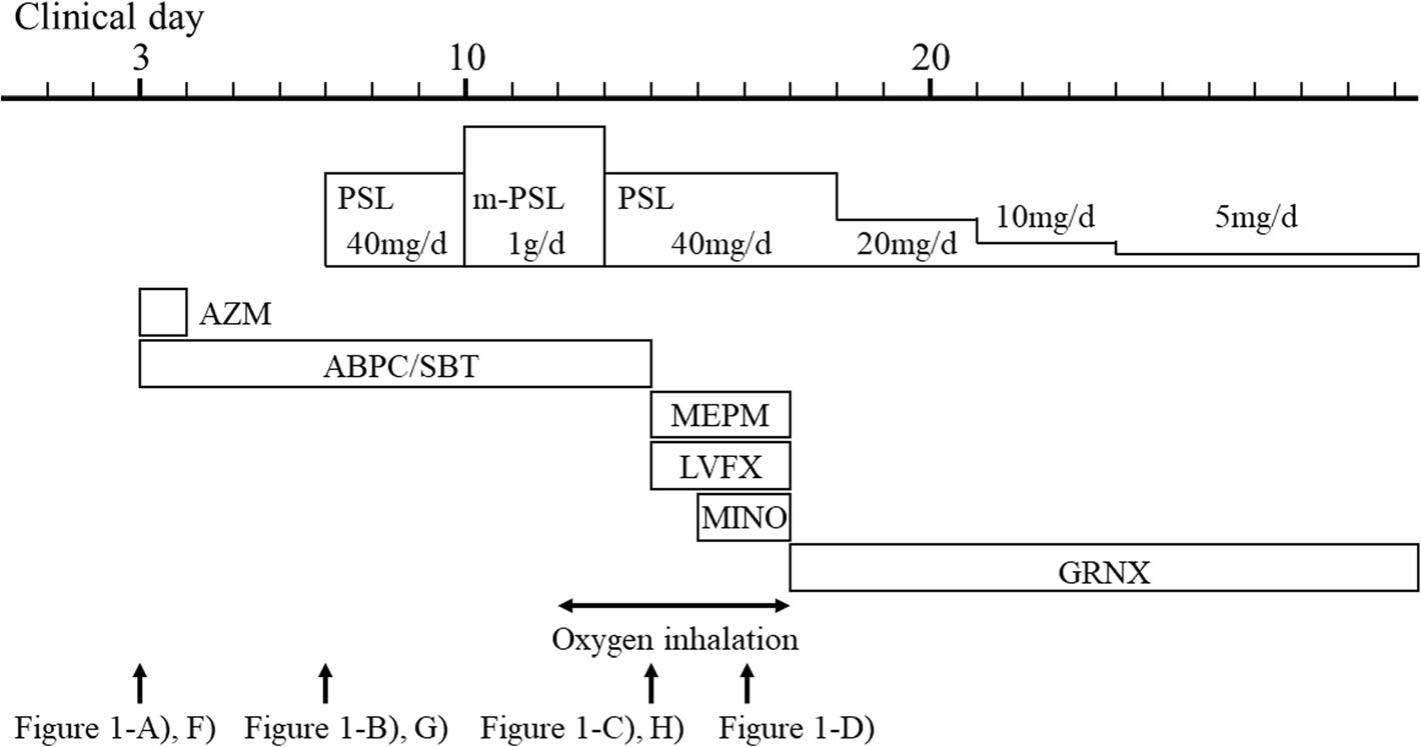
Treatment course. m-PSL: methylprednisolone, PSL: prednisolone, AZM: azithromycin, ABPC/SBT: ampicillin/sulbactam, MEPM: meropenem, LVFX: levofloxacin, MINO: minomycin, GRNX: garenoxacin

A 39-year-old apparently healthy woman complained of fever and productive cough, in March, 2017. Her medical history did not reveal any specific illness, including acquired immune deficiency syndrome, collagen disease, and congenital immunodeficiency. She neither smoked nor consumed alcohol. Three days after onset (clinical day 3), she was admitted to a local general hospital, owing to progressive fever, malaise, and anorexia. On admission, her vital signs were as follows: body temperature, 39.2 °C; blood pressure, 106/64 mmHg; pulse, 80 beats/min with a regular rhythm; SpO_2_, 97% in an air-conditioned room; and respiratory rate, 16 breaths/min. Cyanosis, cardiac murmur, and abnormal breath sounds were absent. The patient’s liver, spleen and lymph nodes were not palpable. Her white blood cell count was 5600/μL, with a shift to the left (81.2% neutrophils). Her aspartate aminotransferase level was 23 IU/L; alanine aminotransferase, 12 IU/L; lactate dehydrogenase, 206 IU/L; and C-reactive protein, 2.4 mg/dL (normal range, 0–0.3 mg/dL). Moreover, the patient’s chest X-ray and chest computed tomography (CT) images revealed subsegmental consolidation in her right lower lobe (Figs. 1a, f). After admission, administration of ampicillin/sulbactam (ABPC/SBT), at 6 g/day, was initiated under a clinical diagnosis of severe community-acquired pneumonia. Azithromycin (AZM) was also given at 2 g/day p.o. stat on clinical day 3 (Fig. 2). Her indirect hemagglutination titer for MP was negative (1:40) on clinical day 4. After admission (clinical day 7), her fever had not subsided, and the pulmonary lesions had extended to the entire right lower lobe as well as to the left lower lobe (Figs. 1b, g). Thus, bronchoalveolar lavage (BAL) and a transbronchial biopsy of the right lower lobe were performed. These examinations revealed nonspecific inflammation with neutrophil infiltration, but no pathogen was identified on pathological or microbiological examination. Contrary to the extremely rapid progression of the pulmonary lesions, her general condition had not significantly deteriorated, which was not compatible with severe bacterial infection. Owing to the unique clinical presentation, organizing pneumonia was considered. Therefore, treatment with prednisolone (PSL) was initiated at 40 mg/day from clinical day 7, and steroid pulse therapy (methylprednisolone 1 g/day) was given on clinical days 10–12. However, neither her symptoms nor her pulmonary lesion improved (Figs. 1 c, h). Rather, her respiratory failure had worsened significantly, after steroid pulse therapy (clinical day 14), as she required oxygen inhalation at 15 L/min on her motion; therefore, she was transferred to our hospital (Hokkaido University hospital) for further examination and treatment. Considering the existence of lower respiratory tract infection due to any rare pathogens, ABPC/SBT was changed to meropenem (MEPM) on clinical day 14, and levofloxacin (LVFX) and minocycline (MINO) were also initiated concurrently. On admission (clinical day 14), her indirect hemagglutination titer for MP had elevated to 1:2560, which was more than that identified on clinical day 4 at the referring hospital. Moreover, BAL fluid (BALF) examination, which was performed on the day of admission to our hospital, using Ribotest™ Mycoplasma (Asahi Kasei Pharma Corporation, Japan), yielded positive results for the mycoplasma antigen. No other pathogen was identified on microbiological examination of the BALF. Based on these clinical findings, we confirmed our case as severe life-threatening MP pneumonia. Thereafter, we continued empirical antibiotic therapy with LVFX, MINO and MEPM, and corticosteroid therapy with PSL at 40 mg/day. Two days later (clinical day 16), her fever, malaise, and hypoxia had resolved, and her pulmonary lesions had significantly improved (Fig. 1d). Therefore, we replaced the antibiotics with garenoxacin (GRNX) as monotherapy, at 400 mg/day, and reduced the dosage of PSL from clinical day 18. The patient was discharged on day 24, and administration of GRNX and corticosteroid therapy were continued until clinical day 30 (Fig. 2). Subsequently, she had an uneventful recovery with no recurrence of fever or pneumonia.

Due to the rarity of our patient’s clinical course, we performed further molecular identification using DNA extracted from her BALF. DNA of MP was identified by real-time polymerase chain reaction (PCR) with Mp181-F and Mp181-R primer pairs and an Mp181-P probe [10]. A previously described RFLP analysis of point mutation in domain V of MP 23SrRNA gene [11] was used to identify mutations known to confer macrolide resistance (2063, 2064, and 2617 in the MP 23S rRNA gene domain V region). The result of the molecular analysis was positive for A2063G mutation, which was, at that time, a common macrolide-resistant mutant of MP in Japan. Based on these results and the clinical course, we confirmed our case as severe MP pneumonia due to a macrolide-resistant strain.

## Discussion and conclusions

In this report, we describe a severe life-threatening case of MPP due to a macrolide-resistant strain. In our case, the initial therapy involving high-dose administration of AZM and corticosteroids was ineffective, and LVFX and MINO were necessary for an improvement in clinical symptoms and reduction in pulmonary lesions. The possibility of bacterial co-infection remained; thus, another antibiotic (MEPM) was administered. However, the clinical course and CT manifestations were not compatible with general bacterial infection, and the repetitively negative bacterial culture from respiratory tract specimens could not fully explain the presence of extensive pulmonary lesions. Hence, we considered that the extreme deterioration noted until clinical day 14 was largely due to macrolide-resistant MPP.

Recently, the prevalence of macrolide-resistant MP isolates has increased worldwide. The most frequent mechanism underlying the resistance is an A-to-G mutation at position 2063 of MP 23S rRNA gene domain V (A2063G), followed by A2063T, A2064G, and A2063C [12]. As previously reported, failure of the initial treatment with macrolides against macrolide-resistant MPP often results in prolonged fever and cough; however, respiratory failure or a fatal course are rare [12–14]. Macrolide-resistant MPP likely did not progress to severe infection owing to its less efficient protein synthesis, caused by a point mutation within its rRNA [15]. Because MP has only one rRNA operon for constructing ribosomes, a point mutation in a macrolide-resistant strain might exclusively affect ribosomal activity.

Some cases of MPP may be life-threatening, involving severe respiratory failure or fatality, and are occasionally defined as fulminant MPP [2–4]. In those life-threatening MPP cases, the average duration from the onset of infection to the development of respiratory failure is reported to be 9–15 days [2]; these cases include those involving deterioration after the administration of certain therapies, such as macrolide or steroid therapy. As respiratory failure development was noted in our case until clinical day 14, we considered that this case was likely of the severe life-threatening/fulminant type.

Recognized as the mechanism and etiology of severe/fulminant MPP, the host’s cellular hyper-immune response to MP is considered to play a central role in disease progression [2]. As MP has no bacterial cell wall, antibiotics that inhibit DNA synthesis, such as macrolides, tetracycline, and fluoroquinolone, are commonly used to treat MP infection. In addition, corticosteroids are broadly recommended for severe/fulminant MPP cases, which present with a hyperactive immune response [2–4]. Izumikawa et al. reported that a relatively high dose of methylprednisolone (> 500 mg/day) combined with appropriate anti-mycoplasma agents effectively improved symptoms in a majority of fulminant MPP cases within 3–5 days [16]. Miyashita et al. recommended the initiation of corticosteroid therapy for severe MPP cases with a serum LDH level above 364 IU/L [17]. However, a definitive treatment for fulminant MPP, in particular that caused by a macrolide-resistant strain, has not been established.

To date, there exist only few reports describing fulminant MPP cases resistant to macrolide and steroid therapy. In a pediatric case, Shen et al. described fulminant MPP due to a macrolide-resistant strain, in which drug-susceptibility was confirmed by cultures [18]. In this case, MPP exacerbated after the administration of AZM and standard-dose methylprednisolone (2 mg/kg/day), and improvement was finally noted after switching antibiotic treatment to moxifloxacin with intravenous immunoglobulin. In an adult case, Kawakami et al. reported that fulminant MPP exacerbated despite the administration of AZM and prednisolone 30 mg/day, but that improvement was noted after minocycline administration [19]. Similar to these cases, our case of MPP also showed rapid improvement after fluoroquinolone and minocycline addition. Overall, we propose that not only hyper-immune activity, but also MP proliferation, may play a critical role in severe/fulminant MPP due to a macrolide-resistant strain. Thus, treatment using fluoroquinolone or tetracycline for suppressing MP proliferation might be indispensable in these cases.

In our case, rapid antigen test was not available in the referring hospital, and definite diagnosis was confirmed on clinical day 14. Although serological antibody test is generally accepted as a standard method for the diagnosis of MPP, it is not suitable for MPP-diagnosis during the acute phase since it requires paired serum samples with a 2–4-week interval [20]. In this situation, immunochromatography-based rapid mycoplasma antigen test, Ribotest™ Mycoplasma, has become available in Japan, since 2013 [21]. This test detects the *M. pnuemoniae* L7/12 ribosomal protein, a component of the 50S ribosome, and its diagnostic sensitivity for MPP has been reported as approximately 60% that of real-time PCR [21]. Until now, the clinical experience and data on Ribotest are still limited only to Japan, and its utility in the management of adult MPP remains unclear. In our case, the Ribotest on BALF performed on clinical day 14 was positive, which proved the existence of a longer-lasting MP pulmonary infection. Since MP has been found to be larger amount in sputum than in upper respiratory tract samples [22, 23], it might be more useful to examine BALF, which is the most directly sampled lower respiratory tract specimen, using Ribotest. At least, had an immediate diagnosis been made for our patient, we could have selected appropriate antibiotics at an earlier stage, which might have achieved more rapid improvement. Considering the difficulty of MPP diagnosis, in particular cases due to macrolide-resistant strains, we suggest that further study might be necessary, which examine the utility of rapid antigen test.

To the best of our knowledge, the present report is the first documented case of severe life-threatening MPP due to a macrolide-resistant strain, which macrolide-resistance was confirmed by genetic analysis. Based on the experience gained from our present case, we suggest that severe MPP due to a macrolide-resistant strain should be considered as a differential diagnosis, when one encounters cases of deteriorating community-acquired pneumonia. This is particularly important when antibiotics other than fluoroquinolone or tetracycline have been administered. The high prevalence of macrolide-resistant MP worldwide should also be recognized, because similar cases of life-threatening MPP may be substantially underdiagnosed.

In conclusion, we report the rare case of severe life-threatening MPP caused by a macrolide-resistant strain in an adult. It highlights the importance of appropriate selection of anti-mycoplasma drugs in the treatment of this condition. In addition, more awareness is needed on the emergence of macrolide-resistant MPP infection, especially in cases where severe infection develops after initial treatment failure.

## Abbreviations

ABPC/SBT: Ampicillin/sulbactam
AZM: Azithromycin
BAL: Bronchoalveolar lavage
BALF: Bronchoalveolar lavage fluid
CT: Computed tomography
GRNX: Garenoxacin
LVFX: Levofloxacin
MEPM: Meropenem
MINO: Minocycline
MP: *Mycoplasma pneumoniae*
PSL: Prednisolone
